# Human Platelets Take up Anti-VEGF Agents

**DOI:** 10.1155/2021/8811672

**Published:** 2021-06-14

**Authors:** B. Sobolewska, B. Fehrenbacher, P. Münzer, H. Kalbacher, S. Geue, Konstantinos Stellos, M. Schaller, F. Ziemssen

**Affiliations:** ^1^Center for Ophthalmology, Eberhard Karls University Tuebingen, Tuebingen, Germany; ^2^Department of Dermatology, Eberhard Karls University Tuebingen, Tuebingen, Germany; ^3^Department of Cardiology and Cardiovascular Medicine, University of Tuebingen, Tuebingen, Germany; ^4^Interfaculty Institute of Biochemistry, Eberhard Karls University Tuebingen, Tuebingen, Germany; ^5^Biosciences Institute, Vascular Biology and Medicine Theme, Newcastle University, Newcastle upon Tyne, UK; ^6^Department of Cardiology, Newcastle Hospitals NHS Foundation Trust, Newcastle upon Tyne, UK

## Abstract

**Purpose:**

Growing evidence suggests different systemic exposure of anti-vascular endothelial growth factor (anti-VEGF) agents with repeated intravitreal application. Since the penetration of anti-VEGF agents through vascular barrier was reported, the interaction of anti-VEGF with nonresident platelets has become a topic of interest. The purpose of this study was to evaluate, with the help of visualization techniques, whether platelets take up the anti-VEGF agents ranibizumab, aflibercept, and bevacizumab.

**Methods:**

The uptake of anti-VEGF agents with or without VEGF treatment was investigated using immunofluorescence and immunogold staining in human platelets. The role of actin filaments and clathrin-coated vesicles in the transport of ranibizumab, aflibercept, and bevacizumab was evaluated by two pharmacologic inhibitors: staurosporine (protein kinase C inhibitor) and cytochalasin D.

**Results:**

All three anti-VEGF agents were taken up by platelets and colocalized with VEGF. Ranibizumab and aflibercept were mainly detected in alpha-granules; however, bevacizumab was equally localized in alpha-granules and in platelet vesicles. Both staurosporine and cytochalasin D completely inhibited the uptake of aflibercept into platelets. Both pharmacological inhibitors also decreased the transport of ranibizumab and bevacizumab into platelets. Bevacizumab was significantly more frequently colocalized within clathrin-coated vesicles than ranibizumab and aflibercept.

**Conclusion:**

All three anti-VEGF agents are taken up by platelets and internalized in alpha-granules, which may result in a higher local exposure of anti-VEGF after the activation of platelets, potentially contributing to arterial thromboembolic events. Clathrin-coated vesicles seem to be more prominent in the transport of bevacizumab than ranibizumab and aflibercept. Nevertheless, whether the different localization and transport of bevacizumab are truly related to specific differences of receptor-mediated endocytosis has to be revealed by further research.

## 1. Introduction

Anti-vascular endothelial growth factor (anti-VEGF) agents have a broad field of application due to their impact on tumor growth and metastasis in oncology or in sealing, and antiangiogenic effect in the treatment of neovascular age-related macular degeneration (nAMD) or other retinal diseases. The inhibitors have a certain range of molecular properties: Ranibizumab is a recombinant humanized 48 kDa antibody fragment (*F*_ab_), designed to easily penetrate the retina with low serum concentrations [1–3]. In contrast, aflibercept is a 115 kDa VEGF receptor fusion protein, which also contains an Fc portion [1, 4]. Similarly, bevacizumab is a full-length recombinant humanized IgG1 antibody and was primarily developed for the intravenous treatment of metastatic cancer [5].

It has been reported several times that monoclonal antibodies are sequestered in platelets, similar to most other serum proteins and growth factors such as platelet-derived growth factors (PDGFs), transforming growth factors (TGFs), vascular endothelial growth factors (VEGFs), and epithelial growth factors (EGFs) [6–8]. Under physiological conditions, platelets circulate in the blood in a resting state and are crucial in detecting vascular injuries to provide hemostasis. Due to contact of platelets with microlesions, platelets adhere to and spread on the thrombogenic matrix forming an activated platelet layer with the subsequent release of proteins from storage granules [9]. Platelet activating factors contribute to the further recruitment and aggregation of platelets, as well as other cells including monocytes and fibroblasts, leading to stabilization of the hemostatic plug [10]. These platelet features have been studied and applied as an autologous platelet concentrate in reconstructive and regenerative medicine (wound healing, maxillofacial bone defect, musculoskeletal soft tissue injuries), as well as in ophthalmology to treat refractive corneal ulcers [10–16] and to increase the closure rate of idiopathic full-thickness macular holes or optic pits after vitrectomy [17]. Activated platelets can also form pathogenic thrombi in patients with atherothrombotic disease [18].

**Figure 1:**
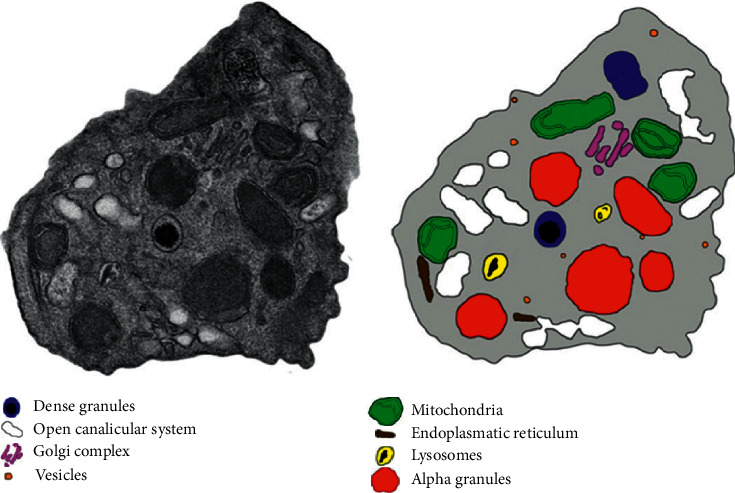
Schematic representation of the platelet organelles: Platelets contain three major granule types: alpha-granules (50–80 per platelet), dense granules (3–6 per platelet), and lysosomes (1–3 per platelet). Platelets also contain open canalicular system, Golgi complex, vesicles, mitochondria, and endoplasmic reticulum.

Until now, the uptake and intracellular transport of anti-VEGF agents have been investigated in retinal endothelium and retinal pigment epithelium; however, little is known about these mechanisms in platelets [6, 19–21]. We have previously shown that increased levels of FITC-labeled ranibizumab, aflibercept, and bevacizumab were found using fluorescence-activated cell sorting (FACS) analysis after the activation of platelets with either thrombin receptor-activating peptide-6 (TRAP), proteinase-activated receptor 4-activating peptide (PAR-4-AP), or thrombin [21, 22]. Therefore, the question arises as to whether all anti-VEGF agents are taken up by platelets and which mechanisms are involved in their endocytosis by platelets (Figure 1).

If platelets act as a “drug vehicle”, anti-VEGF agents may not only target tumor cells, but also contribute to vascular homeostasis and repair after vascular injury [23]. Adverse vascular events such as arterial thromboembolic events (ATEs) have been reported in cancer patients treated with systemic chemotherapy (aflibercept [24], bevacizumab [5, 25, 26]) in comparison to placebo. Moreover, the intravitreal application of anti-VEGF agents, especially aflibercept and bevacizumab, leads to their accumulation in the blood after repeated injections [2]. Therefore, anti-VEGF associated systemic side effects due to increased local concentrations of anti-VEGF agents after platelet activation are of interest, not only in oncology, but also in ophthalmology [11, 27–31].

Considering the fact that platelets play an important role in both vascular homeostasis [32–35] and vascular disease, including atherothrombosis and ATEs [11, 36–39], the purpose of this study was to investigate whether and how platelets take up ranibizumab, aflibercept, and bevacizumab. Furthermore, we investigated whether the cellular uptake differs for the most widely used drugs by characterizing the intracellular trafficking mechanisms.

## 2. Materials and Methods

### 2.1. Isolation of Platelets

Venous blood was drawn into acid-citrate-dextrose (ACD) anticoagulant from healthy Caucasian volunteers, who had not taken any drugs during the previous 10 days. The blood was centrifuged at 200 g for 20 min at room temperature. Platelets were obtained by centrifuging platelet-rich plasma (PRP) at 900 g for 10 min at room temperature and resuspended in Tyrode's buffer (pH 7.4) and 4% formaldehyde, after which 3% paraformaldehyde was added for platelet fixation. This work adhered to the tenets of the Declaration of Helsinki, and the Institutional Ethics Committee of the University of Tübingen granted approval with a waiver of informed consent for this retrospective study using platelet donation of healthy volunteers.

### 2.2. Primary Antibodies

The following primary antibodies were used: FITC-labeled ranibizumab (Lucentis; Novartis Pharma GmbH, Germany), aflibercept (Eylea, Bayer Pharma, Berlin, Germany), and bevacizumab (Avastin; Roche Pharma, Grenzach-Wyhlen, Germany). FITC labeling was performed according to the standard procedures provided by the manufacturer (Sigma-Aldrich, St. Louis, MO, USA). The dilution of anti-VEGF agents was 1 : 100 (10 *μ*g/mL), and rabbit anti-VEGF (Abcam) was 1 : 100.

### 2.3. Confocal Immunofluorescence Microscopy

Platelets were fixed in 4% paraformaldehyde for 3 hours at room temperature and permeabilized with 0.1% Triton-100. After blocking in donkey serum (Sigma), platelets were incubated for one hour with primary and secondary antibodies, respectively (mouse anti-FITC antibody, 1 : 200, Abcam, ab10257; donkey anti-rabbit-DyLight 649, 1 : 800, Dianova, 711-496-152). Finally, platelets were counterstained with phalloidin-Alexa 549 (1 : 100, Molecular Probes, A22283). Moreover, 1% BSA (bovine serum albumin) in PBS (phosphate buffered saline) was used as a control. Immunofluorescence images were examined using a confocal laser scanning microscope (Leica TCS-SP/Leica DM RB confocal laser scanning microscope) and processed with the Leica Confocal Software (LCS) (version 2.61).

### 2.4. Electron Microscopy of Anti-VEGF Agents, VEGF, and Clathrin

Platelets were fixed in 3% paraformaldehyde and 0.01% glutaraldehyde for 3 hours at 4°C and then centrifuged. The pellet was embedded in 3.5% agarose at 37°C and cooled on ice. Small parts of agarose blocks were dehydrated in graded ethanol by gradually lowering the temperature to −35°C and embedded in Lowicryl K4M (Polysciences, Germany) at −35°C. The blocks were cut with an ultramicrotome (Ultracut; Reichert, Vienna, Austria), and ultrathin sections (30 nm) were mounted on formvar-coated nickel grids. After the addition of blocking solution (PBS with 10% goat (Dako)), the ultrathin sections were incubated with primary antibodies overnight and secondary antibody for 1 hour (mouse anti-FITC antibody, 1 : 100, Abcam, ab10257). Washing was done with both 1% BSA in PBS and 0.5% skimmed milk powder diluted in 1% BSA in PBS. Subsequently, the sections were incubated with gold-conjugated goat anti-mouse IgG (gold particle diameter: 6 nm; Jackson) and 12 nm gold-conjugated goat anti-rabbit IgG for 1 hour (gold particle diameter: 12 nm; Jackson) (antibodies were diluted at a ratio of 1 : 20 in PBS/BSA/0.5% skimmed milk powder). Finally, the sections were stained with 1% uranyl acetate for 2 minutes. Samples were examined using a Libra 120 electron microscope (Zeiss Oberkochen) operating at 120 k.

### 2.5. Staurosporine and Cytochalasin D

Staurosporine (protein kinase C (PKC) inhibitor, 10 nM, Sigma-Aldrich, Germany) and cytochalasin D (0.5 mM, Sigma-Aldrich, Germany) were used. The immunocytochemical labeling and silver enhancement (preembedding) were performed on platelets. Subsequently, embedding was performed in glycidyl ether (Serva, Germany). The ultrathin (30 nm) sections were prepared for transmission electron microscopy.

### 2.6. Statistical Analysis

Data of immunogold labeling regarding the localization and colocalization of anti-VEGF agents and VEGF or clathrin are presented as a percentage of gold particles of the total number of gold particles or each compartment. Data are presented as the mean ± SEM (standard error of the mean) of three different experiments. The one-way analysis of variance (ANOVA) was performed between three groups. Significance was considered at *P* < 0.05. Statistical analyses were performed using commercial software (SPSS version 22.0, SPSS, Inc.).

## 3. Results

### 3.1. Intracellular Localization of Anti-VEGF Agents in Platelets

**Figure 2:**
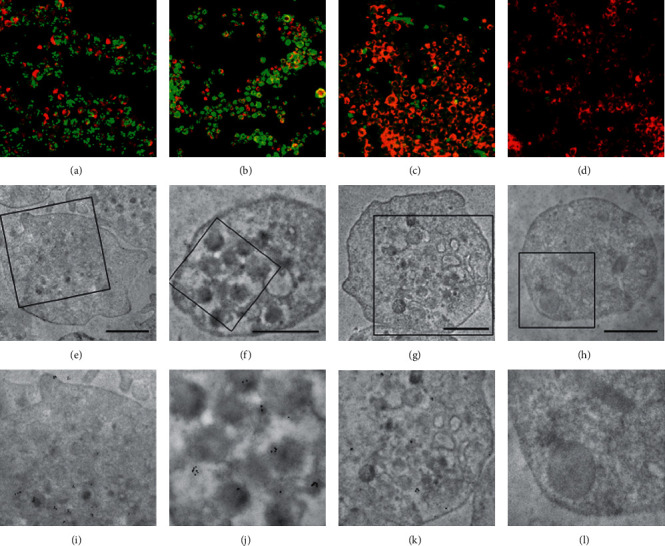
Localization of anti-VEGF agents using immunofluorescence microscopy and immune electron microscopy in human platelets. FITC-labeled anti-VEGF agents (green). Alexa 549-labeled F-actin (red). Overlay of immunofluorescence for anti-VEGF agents and F-actin: (a) ranibizumab, (b) aflibercept, and (c) bevacizumab. (d) Negative control; gold particle (6 nm)-labeled anti-VEGF agents: (e, i) ranibizumab; (f, j) aflibercept; (g, k) bevacizumab; (h., l) negative control. Scale bar represents 1 *µ*m.

All three anti-VEGF agents were taken up to slightly varying degrees by platelets (Figure 2).

**Figure 3:**
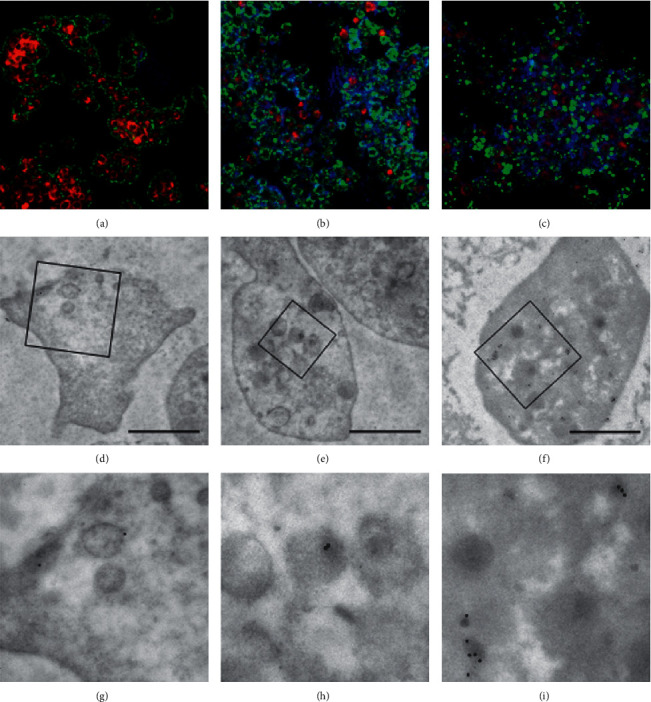
Colocalization of anti-VEGF agents and VEGF using triple immune fluorescence staining and double immunogold staining in human platelets. FITC-labeled anti-VEGF agents (green) and VEGF (blue); Alexa 549-labeled F-actin (red). Overlay of immunofluorescence for anti-VEGF agents, VEGF, and F-actin: (a) ranibizumab, (b) aflibercept, and (c) bevacizumab. Large gold particle (12 nm)-labeled VEGF and small gold particle (6 nm)-labeled anti-VEGF agents: (d, g) ranibizumab; (e, h) aflibercept; (f, i) bevacizumab. Scale bar represents 1 *µ*m.

Immunogold microscopy confirmed that anti-VEGF agents were present in resting platelets following a two-hour coincubation. Both ranibizumab and aflibercept were present in platelets (Figure 2) and colocalized with VEGF in alpha-granules (Figure 3).

**Figure 4:**
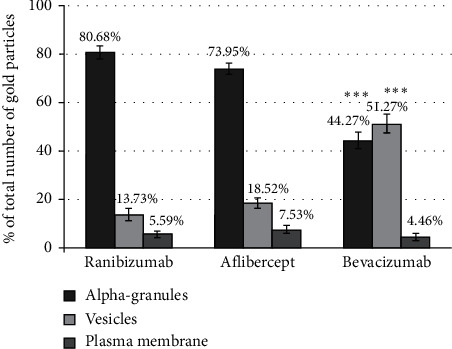
Distribution of anti-VEGF agents in different platelet fractions: quantification of 850 gold particles, 35 randomly selected platelet cross-sections positive for ranibizumab, aflibercept, and bevacizumab. The data represent three experiments. The number of gold particles corresponding to each anti-VEGF agent is expressed as the percentage of the total number of gold particles. Significance of differences between the anti-VEGF agents was calculated by one-way ANOVA and indicated by asterisks with ^*∗∗∗*^*P* < 0.001.

Quantitative analysis of gold staining revealed that 80.68 ± 2.68% of ranibizumab and 73.95 ± 2.33% of aflibercept were contained in alpha-granules (*P*=0.03). In addition, 51.27 ± 3.88% and 44.27 ± 3.39% of bevacizumab were contained in vesicles and in alpha-granules (*P*=0.0001 between all anti-VEGF agents) (Figure 4).

All VEGF-inhibitors colocalized with VEGF, with 41.34 ± 1.76% and 41.15 ± 2.53% of alpha-granules labeled for ranibizumab or aflibercept and VEGF, respectively (*P*=0.81). Bevacizumab showed colocalization with VEGF to an extent of 70.38 ± 2.70% (*P*=0.0001 between all anti-VEGF agents).

### 3.2. Effect of Staurosporine and Cytochalasin on Transport of Anti-VEGF Agents into Platelets

**Figure 5:**
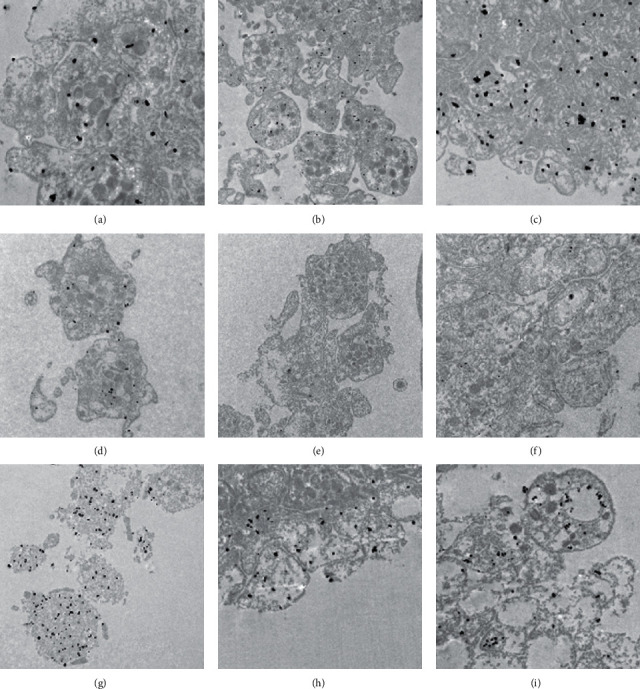
Silver enhancement (preembedding) using immunoelectron microscopy in human platelets exposed to pharmacologic inhibitors: staurosporine and cytochalasin D. Gold particle (1 nm)-labeled anti-VEGF agents: (a) ranibizumab, (b) aflibercept, and (c) bevacizumab. (a, b, c) Platelets without exposure to pharmacologic inhibitors. (d, e, f) Platelets exposed to staurosporine. (g, h, i) Platelets exposed to cytochalasin D. Scale bar represents 1 *µ*m.

**Figure 6:**
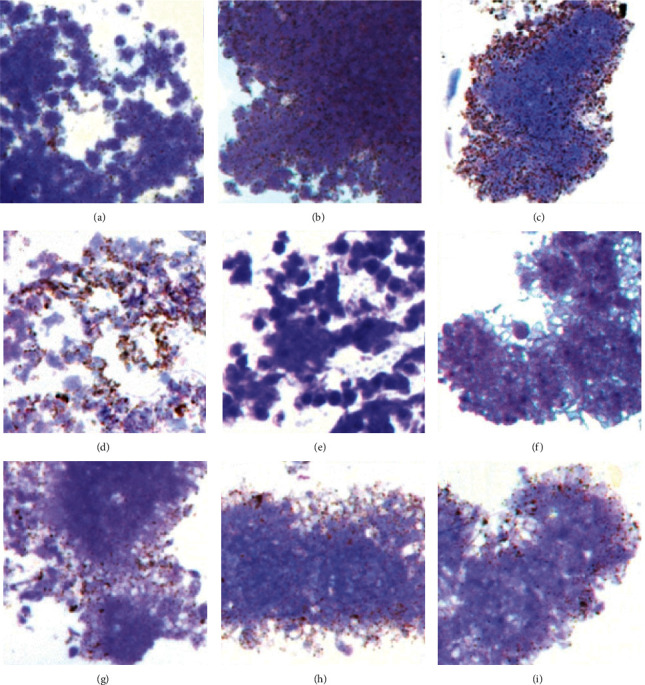
Immunogold staining using immunoelectron microscopy in human platelets exposed to pharmacologic inhibitors: staurosporine and cytochalasin D. Gold particle (1 nm)-labeled anti-VEGF agents: (a) ranibizumab, (b) aflibercept, and (c) bevacizumab. (a–c) Platelets without exposure to pharmacologic inhibitors. (d–f) Platelets exposed to staurosporine. (g–i) Platelets exposed to cytochalasin D. Scale bar represents 1 *µ*m.

The nonselective inhibition of protein kinases, including protein kinase C by staurosporine or stopping actin polymerization by cytochalasin D, completely inhibited the transport of aflibercept into platelets. Both pharmacological inhibitors also decreased the transport of bevacizumab into platelets. Protein kinase C inhibition by staurosporine impaired the transport of bevacizumab to a lesser extent than ranibizumab. In the platelets exposed to cytochalasin D, the transport of ranibizumab was unchanged in comparison to the control (Figures 5 and 6).

### 3.3. Colocalization of Anti-VEGF Agents and Clathrin

**Figure 7:**
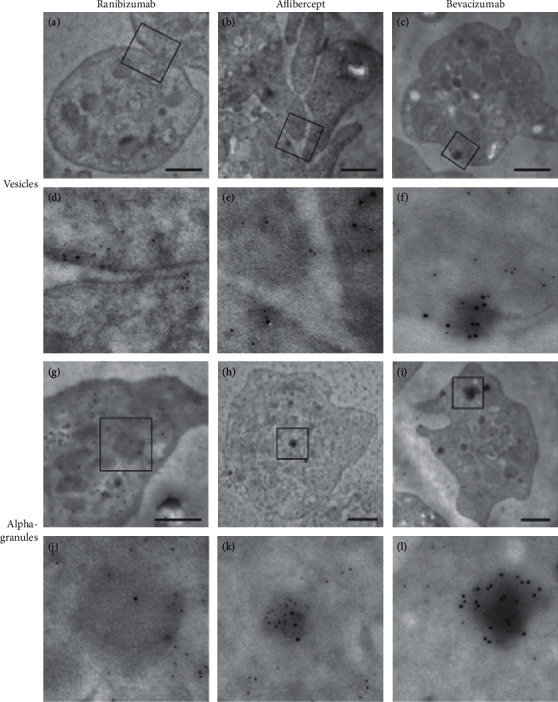
Colocalization of anti-VEGF agents and clathrin using double immunogold staining in vesicles (a–c) and alpha-granules (d–f) of human platelets. Large gold particle (12 nm)-labeled anti-VEGF agents and small gold particle (6 nm)-labeled clathrin: (a, g, d, j) ranibizumab; (b, h, e, k) aflibercept; (c, i, f, l) bevacizumab. Scale bar represents 1 *µ*m.

Quantitative analysis of gold staining showed that ranibizumab, aflibercept, and bevacizumab colocalized with clathrin in 25.49 ± 2.33%, 18.21 ± 2.68%, and 43.56 ± 3.88%, respectively (*P*=0.0001 between all anti-VEGF agents). In particular, at the periphery of vesicles, an intensive accumulation of bevacizumab in the vicinity of clathrin signals was observed (Figure 7).

## 4. Discussion

Anti-VEGF agents are widely used in treatment of cancer patients and are the first-line therapy of neovascular AMD and macular edema secondary to diabetes and retinal vein occlusion. Therefore, the comparison between compounds is warranted, and the safety profile has gained interest since vascular events were observed in a recently approved drug [40, 41]. The adverse events are primarily explained by the blocking of VEGF signaling and the different suppression of serum proteins by anti-VEGF agents [3, 42, 43]. Meyer et al. showed a possible molecular mechanism of bevacizumab-induced platelet activation by the cross-linking of Fc receptors [44], which was confirmed by Nomura et al. [45]. In the previous experiments, we observed that activation-dependent platelet function is more impaired with aflibercept and bevacizumab compared to ranibizumab, without any impact on platelet aggregation. Moreover, FITC-labeled aflibercept and bevacizumab, as well as ranibizumab, were significantly upregulated in activated platelets [21, 22]. Therefore, the anti-VEGF agents might be transported into platelets and then localized in one of three major granule types: *α*-granules, dense granules, and lysosomes; this can be demonstrated by immunofluorescence and electron microscopy [46–49].

In the current study, we were able to confirm and further characterize the uptake of three anti-VEGF agents into platelets. Ranibizumab, aflibercept, and bevacizumab were localized in alpha-granules. However, bevacizumab was equally found in both alpha-granules and platelet vesicles. The bevacizumab results are in accordance with the study of Verheul et al., which showed the colocalization of bevacizumab with P-selectin and fibronectin to be indicative of alpha-granules [6]. However, electron microscopy in this study provided further information about potential transport processes. Immunogold staining confirmed that bevacizumab was equally localized both in alpha-granules and in platelet vesicles, in contrast to ranibizumab and aflibercept. In addition, bevacizumab was colocalized with VEGF at a significantly higher level than ranibizumab and aflibercept despite the same concentration of anti-VEGF agents used in our experiment. Systemic exposure to bevacizumab following intravitreal administration is assumed to be much higher and longer in comparison to the two other anti-VEGF agents [3], which could be explained by the release of bevacizumab into platelet extracellular vesicles by activated platelets.

As already pointed out by Berezin et al. and Gasecka et al., platelets are the main source of extracellular vesicles in plasma, which are the cargo for a large number of biological-active molecules including cytokines, chemokines, hormones, enzymes, growth factors, and their receptors (e.g., VEGF), as well as adhesion receptors coordinating cell-to-cell interactions [50, 51]. They contribute to numerous biological mechanisms such as microvascular inflammation, atherosclerotic plaque shaping and rupture, endothelial dysfunction, angiogenesis, neovascularization, thrombosis, cardiac remodeling, and kidney dysfunction [52–57]. In addition, platelet extracellular vesicles seem to be associated with increased blood thrombogenicity and the subsequent risk of atherothrombotic events since they were shown to adhere to the injured endothelium and recruit activated platelets [58–60].

The question of whether platelet extracellular vesicles as a potential marker of “vulnerable blood” might be helpful in identifying patients at risk for adverse events has been raised. However, further research is needed to develop and standardize the current methods for the accurate determination and quantification of platelet extracellular vesicles [55, 61–63].

In other studies on the uptake of anti-VEGF drugs, bevacizumab was mainly found close to or attached to the cytoskeleton, along actin filaments, but also in early endosomes in retinal pigment epithelial (RPE) cells [64, 65]. In contrast, ranibizumab was not observed within the cytoskeletal fraction [65]. In addition, aflibercept uptake into RPE as well as retinal endothelial cells (iBREC: immortalized bovine retinal endothelial cells) was reported to lead to localization close to the Golgi apparatus [66–68]. The different localization of bevacizumab points strongly to the receptor-mediated endocytosis of bevacizumab. Since bevacizumab, but not ranibizumab, colocalized with actin filaments in RPE cells [64, 65], two pharmacological inhibitors of the endocytic pathway [69], cytochalasin D and staurosporine, were evaluated in this study. Both pharmacologic inhibitors disrupted actin polymerization and totally inhibited the endocytosis of aflibercept into platelets. Actin-mediated transport has a greater significance for phagocytosis/macropinocytosis [70, 71]. In platelets pretreated with staurosporine or cytochalasin D, a decrease of intracellular bevacizumab was observed in accordance with the investigations of Terasaki et al. [68]. Although no significant inhibition of bevacizumab uptake was found, receptor-mediated endocytosis cannot be ruled out since actin-disrupting agents do not eliminate all actin structures [71]. Moreover, actin filaments do not seem to play a main role in receptor-mediated endocytosis [71–73]. Further, staurosporine inhibited the transport of ranibizumab to a greater extent than bevacizumab in our study, and cytochalasin D did not change its transport into platelets. The discrepancy in effect between these two pharmacological inhibitors of ranibizumab might be related to disrupted cell structure or platelet aggregation.

The presence of bevacizumab in platelet vesicles and the reduced uptake of anti-VEGF agents after inhibition with actin-disrupting agents drew our attention to the role of one of the most important receptor-mediated endocytic pathways, which involves clathrin-coated pits [73]. Since clathrin is a major component of coated vesicles, electron microscopy allowed us to visualize the colocalization of anti-VEGF agents and clathrin [74], which was significantly more frequently observed with bevacizumab than with ranibizumab and aflibercept. This proved the presence of a clathrin-dependent endocytosis of anti-VEGF agents in platelets. Furthermore, this observation suggests Fcɣ-receptor-dependent endocytosis of bevacizumab [75–77]. Since ranibizumab is a Fab fragment of monoclonal antibody, clathrin-dependent endocytosis could also be caused by binding to the VEGF receptor [78–81]. Nevertheless, the extent of Fc receptor endocytosis in uptake and trafficking of bevacizumab or aflibercept is still unclear and has to be elucidated in future studies.

In conclusion, all three anti-VEGF agents were taken up by platelets, mainly via clathrin-dependent endocytosis. Ranibizumab, aflibercept, and bevacizumab were localized in alpha-granules. Therefore, they are transported in platelets and may be released with pro- or antiangiogenic proteins from platelets at different platelet activation sites. In contrast to ranibizumab and aflibercept, bevacizumab was equally found in alpha-granules and in platelet vesicles resembling endosomes, which is consistent with its more frequent colocalization with clathrin; therefore, it is evidence for receptor-mediated endocytosis. The binding of intravenously administered bevacizumab to the Fc receptor could lead to an increased risk of ATEs due to its accumulation in platelets through sorting away from the degradation pathway [2, 82]. Therefore, deviating endocytosis pathways and localization in the cells may be manifested in different pharmacological activities and the safety profile of anti-VEGF agents. Further research is needed to show the relevance of platelet-loaded anti-VEGF agents in vascular healing after injury and thromboembolic events.

## Data Availability

The data used to support the findings of this study are available from the corresponding author upon request.
